# Accuracy of CBCT and dental-MRI for detecting peri-implant bone defects at single titanium implants: an *in-vitro* pilot study

**DOI:** 10.1186/s40729-026-00709-y

**Published:** 2026-08-19

**Authors:** Katharina Hartung, Nikolai Miotk, Arne Lauer, Franz Sebastian Schwindling, Mathias Nittka, Ali Özen, Ute Ludwig, Jan-Bernd Hövener, Michael Bock, Tabea Flügge, Anne-Katrin Eisenbeiss, Peter Rammelsberg, Sabine Heiland, Martin Bendszus, Tim Hilgenfeld

**Affiliations:** 1 Department of Neuroradiology, Heidelberg University Hospital, Im Neuenheimer Feld 400, 69120 Heidelberg, Germany; 2 Department of Prosthetic Dentistry, Medical University of Innsbruck, Innsbruck, Austria; 3 Division of Medical Physics, Department of Diagnostic and Interventional Radiology, University Medical Center Freiburg, Faculty of Medicine, University of Freiburg, Freiburg, Germany; 4 Department of Radiology and Neuroradiology, University Medical Center Schleswig-Holstein, Kiel University, Kiel, Germany; 5 Research & Clinical Translation, Magnetic Resonance, Siemens Healthineers AG, Erlangen, Germany; 6 Department of Oral and Maxillofacial Surgery, University Medical Center Schleswig-Holstein, Christian-Albrechts University, Kiel, Germany; 7 Department of Prosthodontics, Heidelberg University Hospital, Heidelberg, Germany; 8 Department of Oral and Maxillofacial Surgery, Charité – Universitätsmedizin Belin, corporate member of Freie Universität Berlin and Humboldt Universität Zu Berlin, Hindenburgdamm 30, 12203 Berlin, Germany

**Keywords:** Dental implants, Peri-implantitis, CBCT, MRI

## Abstract

**Purpose:**

To evaluate the reliability and accuracy of optimized dental-MRI (dMRI) for detecting and classifying peri-implant bone defects around single titanium implants.

**Methods:**

48 titanium implants were inserted into bovine ribs. 24 implants had surrounding standardized defects (1-wall, 2-wall, 3-wall, 4-wall) in two sizes (1 mm and 3 mm), while the other 24 implants showed no peri-implant bone defects. CBCT and dMRI were performed and analyzed twice by two readers. 3 Tesla MRI was conducted using an innovative intraoral coil and a novel, T1-weighted sequence for metal artifact reduction (CS-SEMAC). Cohen's Kappa (κ), sensitivity, and specificity were assessed for bone defect detection, size, and type. Modality differences were evaluated using the exact McNemar test and Firth penalized-likelihood logistic regression.

**Results:**

For bone defect detection, almost perfect intra-/inter-rater reliability (*κ-*value range: 0.958–1) was found for both imaging modalities, with high sensitivity/specificity (CBCT 100%/100%, 95% CI 0.962–1.000/0.962–1.000; MRI 98%/100%, 95% CI 0.927–0.998/0.962–1.000), confirmed by Firth regression (*p* = 0.22). For defect type classification, both CBCT (*κ-*value range: 0.884–0.913) and MRI (*κ*-value range: 0.794–0.942) demonstrated almost perfect intra-/inter-rater reliability. Overall sensitivity/ specificity were 86%/99% (95% CI 0.780–0.926 and 0.985–0.995) for CBCT and 84%/99% (95% CI 0.755–0.910 and 0.985–0.995) for dMRI, with no significant difference between modalities (Firth-adjusted OR 0.85, 95% CI 0.38–1.88, *p* = 0.68).

**Conclusions:**

Under optimized ex vivo conditions, dMRI offers a promising reliability and accuracy for identifying and classifying peri-implant bone defects around single titanium implants. High-resolution dMRI facilitates radiation-free, three-dimensional peri-implant tissue visualization.

## Background

As dental implants become increasingly popular with a projected compound annual growth rate of 8%, the prevalence of peri-implant diseases is rising, making early and accurate detection of these conditions more critical than ever [1, 2]. The overall prevalence of peri-implantitis is estimated to be 22%, while around 43% of patients with implants suffer from peri-mucositis [3]. Peri-implantitis is an inflammatory disease caused by bacterial biofilms affecting both soft and hard tissues, leading to three-dimensional bone loss around the implant. This results in a loss of osteointegration and, ultimately, implant failure [4, 5].

Titanium remains the material of choice for dental implants, owing to its excellent biocompatibility, corrosion resistance, high strength-to-weight ratio, and good osteointegration properties [6]. Currently, the gold standard for imaging peri-implant disease is intraoral radiography (IR), which offers high hard-tissue contrast and is effective for detecting peri-implant bone lesions [5, 7]. Although IR excels at visualizing vertical bone defects and peri-implant bone loss in the mesial and distal directions, it struggles with assessing the buccal and lingual aspects and circumferential defects due to the limitations of two-dimensional imaging, potentially leading to underestimation of peri-implant bone loss [8–10]. This is particularly relevant because initial peri-implant bone loss is most commonly observed on the buccal and lingual sides of the implant [11–13].

Cone beam computed tomography (CBCT), while offering three-dimensional imaging with high spatial resolution, has other challenges, including higher radiation exposure and beam hardening artifacts due to dental restorations and replacement material that lead to overestimation of bone defect size around titanium implants [12, 14, 15]. These artifacts limit accurate peri-implant assessment [11, 13]. Given that the configuration of peri-implant bone defects plays a critical role in clinical outcomes, achieving high diagnostic accuracy is of paramount importance [16, 17].

Dental magnetic resonance imaging (dMRI) offers superior soft-tissue contrast without ionizing radiation, making it a promising imaging modality. Previous studies have highlighted its applicability for implant planning [18] and its capabilities for identifying and quantifying peri-implant defects around ceramic implants [19, 20]. However, visualizing peri-implant tissues around titanium implants with MRI, has proven difficult due to extensive metal-induced artifacts within a radius of up to 2 cm around the implants [9, 21, 22]. Using a combination of novel MRI sequences for metal artifact reduction and novel intraoral coil developments, we assessed the feasibility of dMRI to accurately and reliably detect and correctly classify peri-implant bone defects around titanium implants compared to CBCT.

## Methods

The prospective, diagnostic accuracy study was conducted in compliance with the STARD Guidelines [23].

Fresh bovine ribs were dissected to remove all soft tissues and subsequently cut into 48 pieces, each approximately 4 cm in length. No animals were sacrificed specifically for this study. The bone samples were obtained from animals processed at a local slaughterhouse. The specimens were then randomly assigned into two groups: a control group (n=24) and a study group (n=24), with the latter group receiving bone defects around the implants. Given the pilot nature of the study, the sample size was selected based on recommended reference ranges for pilot studies [24–26]. Peri-implant bone defects of two sizes (depth/length: 1 mm and 3 mm) were created using round diamond burs (coarse grit, 151 µm) in two sizes (1-mm defect: Ref. No. 6801.314.010 VPE 5.

3-mm defect: Ref. No. 6801.314.029 VPE 5; Komet Brasseler, Lemgo, Germany) [5]. The thickness of the bone samples was adjusted to 6.1 mm for those with small defects and 10.1 mm for those with large defects. Defect morphology was classified into one-wall (1w), two-wall (2w), three-wall (3w), and four-wall (4w) categories, following the classification by Renvert and Giovannoli [27]. For example, a three-wall defect is characterized by the presence of three remaining bony walls and the absence of one wall. Each defect morphology was therefore represented in three specimens for each defect size.

To create a rough biological bone surface of peri-implant disease, the bone samples were soaked in 70% perchloric acid for four hours. To limit the acid etching to the implant area, the implant bed was then sealed with dental wax. Before implant insertion, CBCT imaging was performed on the specimens to serve as a baseline reference for defect classification, free from artifacts, and to detect any pre-existing bone defects.

A single titanium implant (Roxolid®, Bone Level Implant, SLActive® 10 mm, diameter: 4.1 mm RC, with a RC Variobase® for crown, Institut Straumann AG, Basel, Switzerland) was inserted into each bone specimen. Implantation followed the manufacturer’s guidelines.

Post-implantation imaging was carried out using both dMRI and CBCT. For CBCT (3D Accuitomo 170, J Morita; Kyoto, Japan), the following parameters were applied: 4 × 4 cm^2^ field of view (FOV), 4 cm FOV height, 90.0 kV voltage, 5.0 mA tube current, 14-bit depth, 360° rotation over 30.8 s, 501 frames, and isotropic voxel size of 80 μm, with a slice interval of 0.480 mm and slice thickness of 0.480 mm. To realistically simulate scattering effects caused by the soft and hard tissues surrounding the implant, the specimens were surrounded by a human mandible and placed inside a cylindrical acrylic resin phantom filled with water, as previously described [28].

**Fig. 1:**
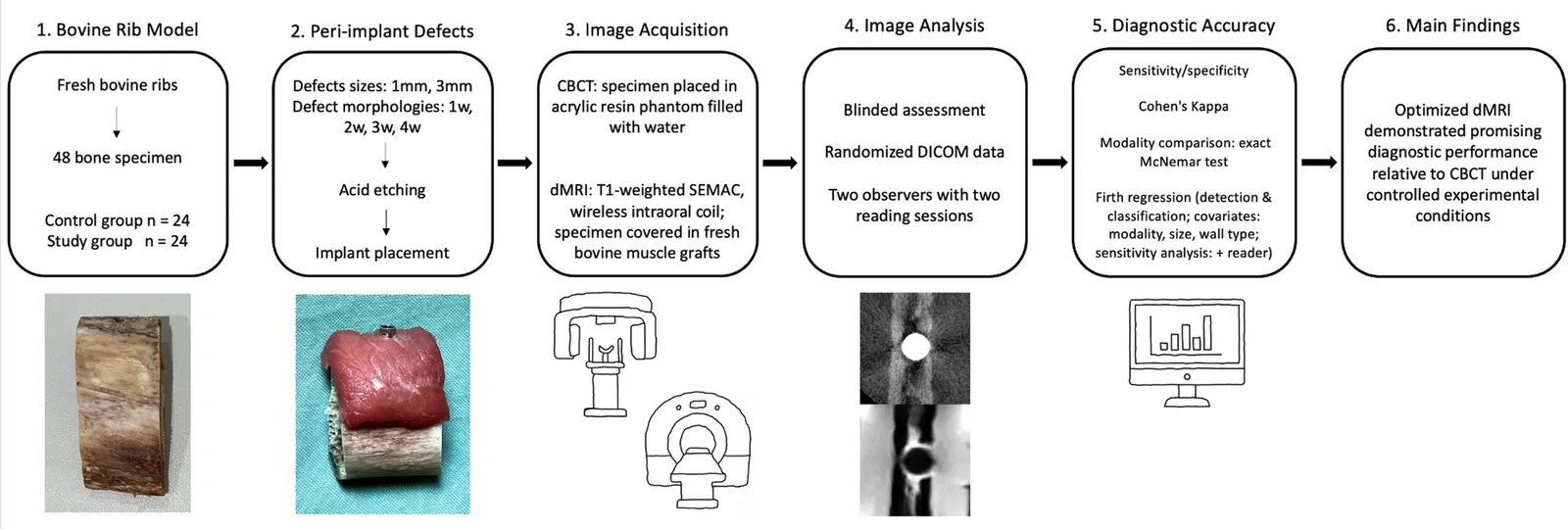
Workflow design

For dMRI, a 3 Tesla MRI system (MAGNETOM Prisma fit; Siemens Healthineers, Forchheim, Germany) was used in combination with a novel, single-channel wireless intraoral coil [29]. The intraoral coil was coupled to a small 40-mm surface receiver coil (Loop4, Siemens Healthineers, Forchheim, Germany). The intraoral coil system comprised two 2-cm-diameter loops with a resin coating and a 1.7 cm gap between them. A variable capacitor was added for tuning to the Larmor frequency at 3 Tesla (123.24 MHz), and crossed diodes were inserted for passive detuning during radio-frequency excitation. To simulate soft tissues, the implant sites were covered with fresh bovine muscle grafts (Fig. 1). Previous studies have shown that infection-related soft tissue changes, such as edema, can be distinguished from healthy muscle, indicating that our model does not artificially enhance dMRI diagnostic performance [30, 31].

To reduce both in-plane and through-plane distortions due to metal artifacts as well as achieve a high spatial resolution, a T1-weighted SEMAC sequence with compressed sensing was employed [32, 33]. The sequence parameters were: repetition/echo time = 793/20 ms; field of view 140 × 96 mm^2^; in-plane resolution 0.31 mm × 0.31 mm; slice thickness = 0.7 mm; acquisition matrix = 448 × 308; number of averages = 1; spacing between slices = 0.0 mm; pixel bandwidth = 585 Hz/pixel; flip angle = 150°; phase encoding direction = right-to-left; turbo factor = 5; SEMAC = 20 slice-encoding steps; acquisition time = 04:14 min. Images were acquired in three orthogonal planes.

**Fig. 2:**
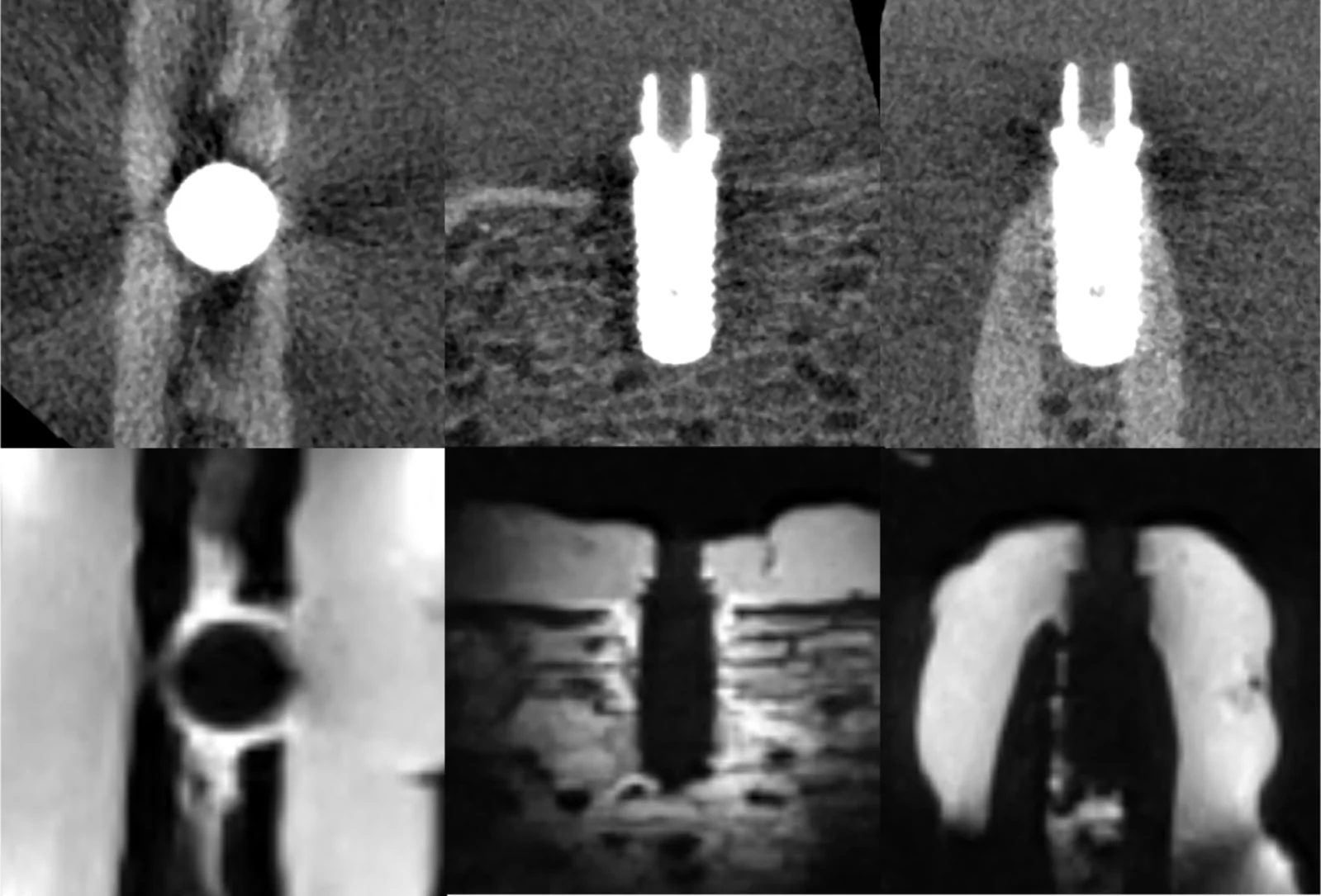
Examples of small 3-wall defects in CBCT/dMRI (upper/lower row) in axial, sagittal, and coronal reconstruction (left to right)

**Fig. 3:**
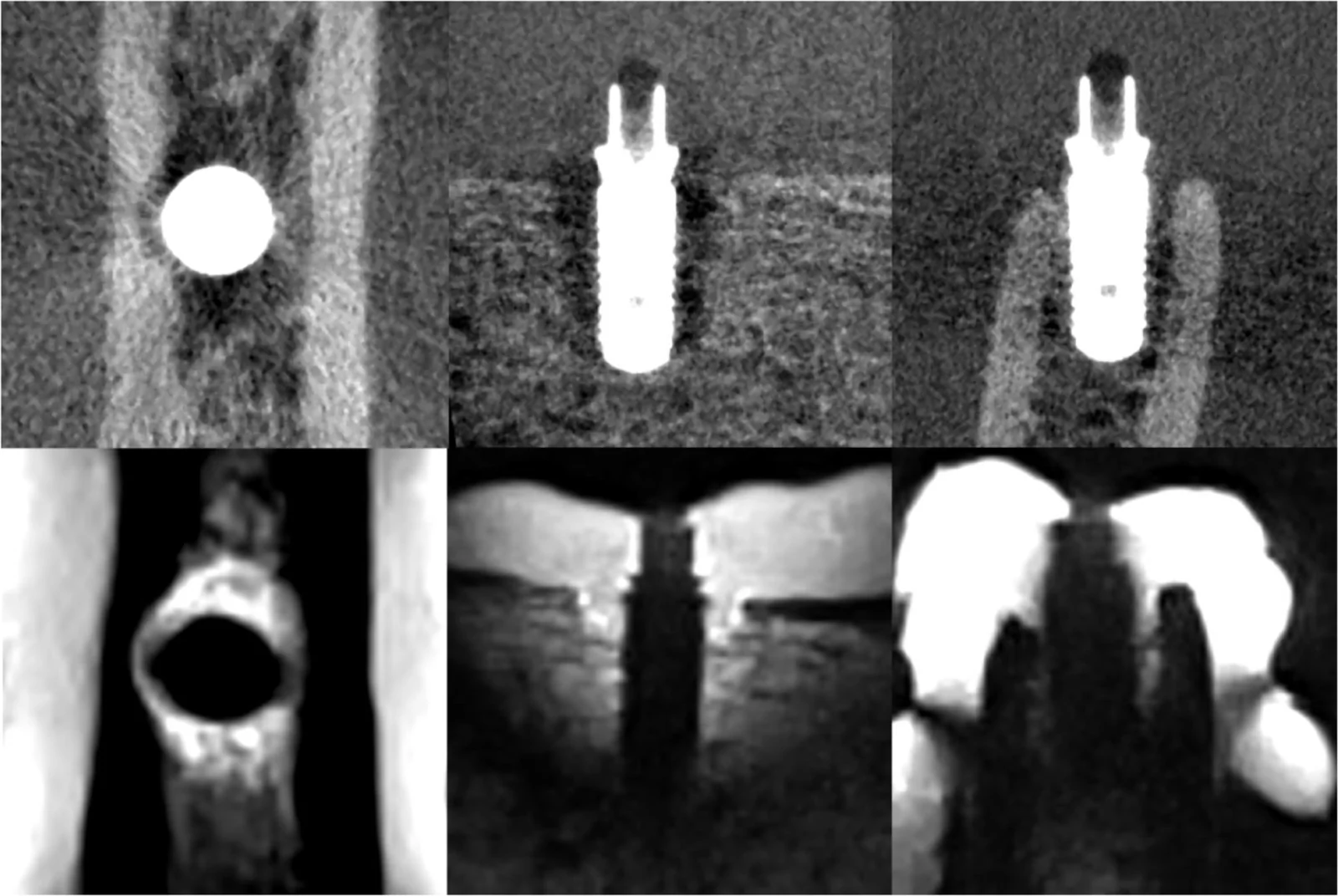
Examples of large 4-wall defects in CBCT/dMRI (upper/lower row) in axial, sagittal, and coronal reconstruction (left to right)

The acquired images were exported as DICOM files and randomized using randomizer.com [34]. Image analysis was performed using OsiriX DICOM Imaging Software (Version 11.0, Geneva, Switzerland) on multiplanar reconstructions, with datasets analyzed in a random order, which was alternated for the second run of analysis. Two radiologists with three and more than ten years of experience in dental imaging independently performed the analysis. Observers were allowed to scroll through the datasets and adjust the contrast. To minimize confounding factors, the analysis was carried out under identical lighting conditions and using the same computer system. The datasets were evaluated for the presence of peri-implant bone defects, as well as defect size (small versus large) and morphology (1w versus 2w versus 3w versus 4w; Figs. 2 and 3). To avoid learning bias, the evaluations were repeated with a minimum two-week interval between assessments.

Statistical analysis was conducted using SPSS Statistics Software (IBM; Version 28.0) and GraphPad Prism Software (GraphPad Software, Inc.; Version 10.0) and Rstudio (Posit Software, PBC, Version 2026.05.1+225) with the *logistf* package. Cohen’s Kappa (κ) was used to determine intra- and inter-rater agreement. The κ-values were interpreted according to Landis and Koch [35]. For diagnostic accuracy, sensitivity and specificity were calculated for each defect size, defect type, and imaging technique, with exact (Clopper-Pearson) 95% confidence intervals; 95% confidence intervals were also calculated for Cohen's Kappa. Differences between imaging modalities in defect detection were assessed using the exact McNemar test. Modality differences were additionally evaluated using Firth penalized-likelihood logistic regression, for defect detection, and, for defect classification accuracy (defined as correct assignment of both defect size and wall count in a correctly detected defect), with imaging modality, defect size, and wall type as covariates and reader added as a covariate in a sensitivity analysis. Wall type was modeled as a nominal factor, with an ordinal specification examined secondarily. The significance level was set at *p* < 0.05. All *p*-values are descriptive and should be interpreted as exploratory.

## Results

### Assessment of reliability

**Fig. 4:**
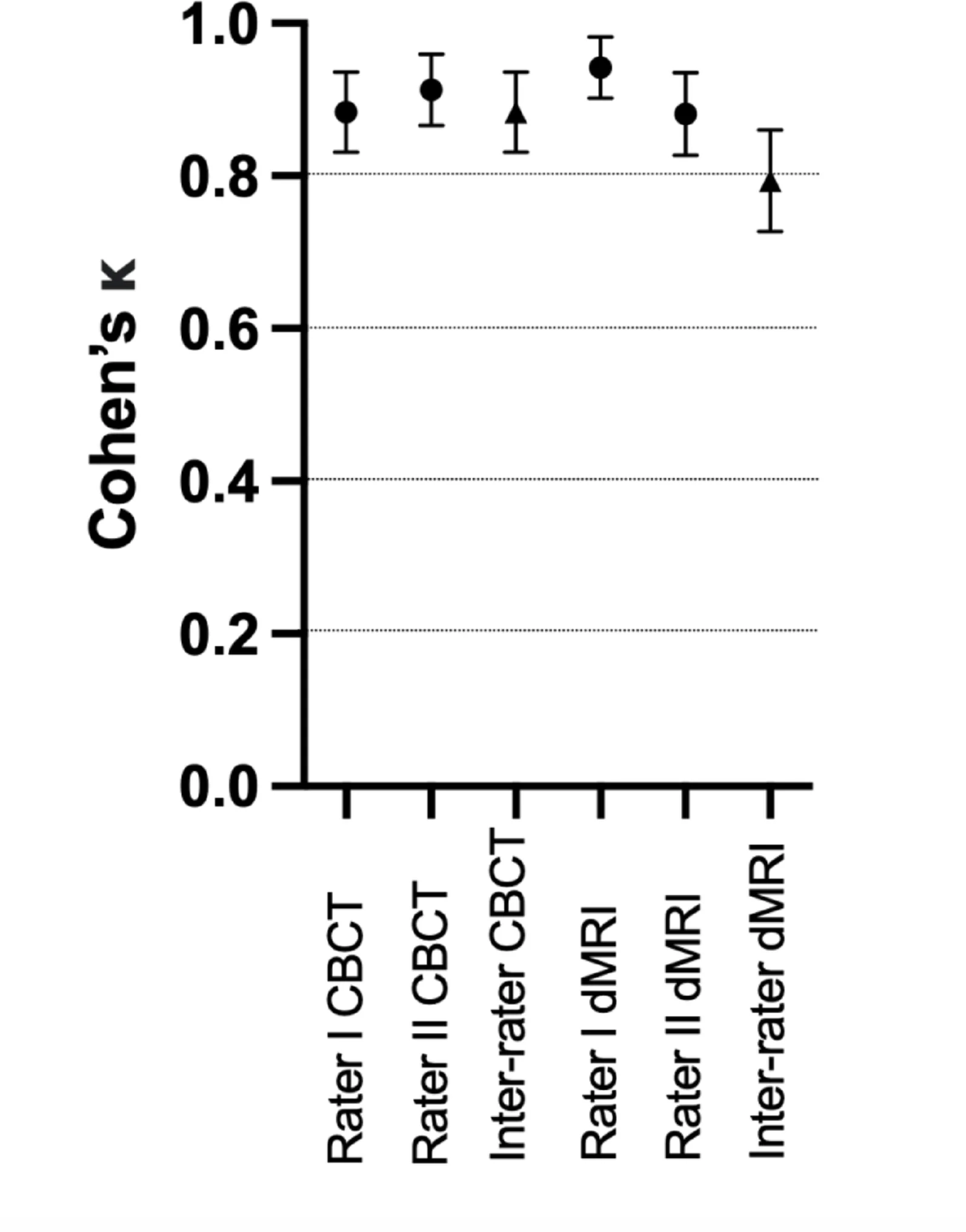
Intra- and inter-rater agreement with standard error of the mean (SEM) for defect type classification in dMRI and CBCT

The intra- and inter-rater reliability for defect detection in dMRI and CBCT was almost perfect for both raters. For dMRI, intra-rater agreement was κ = 1.00 (no discordant readings) for both raters, and inter-rater agreement was κ = 0.958 (95% CI 0.878–1.000). For CBCT, intra-rater agreement was κ = 1.00 (no discordant readings) for both raters, and inter-rater agreement was κ = 1.00 (no discordant readings). For defect type classification, the intra- and inter-rater reliability ranged from substantial to nearly perfect (Fig. 4). Intra-rater agreement ranged from κ = 0.881 (95% CI 0.774–0.987) to κ = 0.942 (95% CI 0.864–1.000) for dMRI and from κ = 0.884 (95% CI 0.780–0.988) to κ = 0.913 (95% CI 0.820–1.000) for CBCT. Inter-rater agreement was κ = 0.794 (95% CI 0.664–0.925) for dMRI and κ = 0.884 (95% CI 0.780–0.989) for CBCT.

### Diagnostic accuracy

Results showed that both dMRI and CBCT performed well in classifying defects, with generally high sensitivity and specificity across all defect types. For defect detection sensitivity and specificity were 100%/100% (95% CI 0.962–1.000 and 0.962–1.000) for CBCT and 97.9%/100% (95% CI 0.927–0.998 and 0.962–1.000) for dMRI. The two modalities disagreed on only 2 of 96 matched positive reading-pairs, in both instances a single small, three-walled specimen that was missed on dMRI. No false-positive calls occurred in either modality (exact McNemar *p* = 0.500). Because this near-perfect classification produced complete separation, modality was additionally compared using Firth penalized-likelihood logistic regression, which confirmed no significant difference in detection (odds ratio [OR] for dMRI vs. CBCT 0.20, 95% CI 0.001–2.45, *p* = 0.22).

**Fig. 5:**
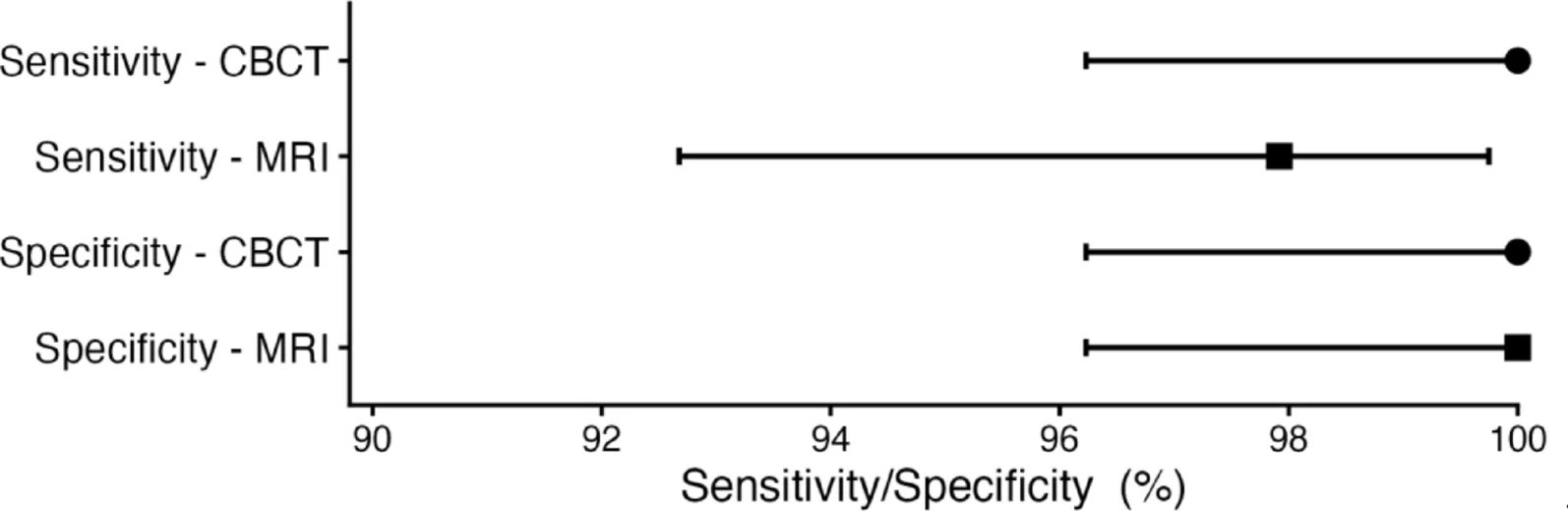
Sensitivity and Specificity (%) of CBCT and dMRI for detection and correct classification for all specimen. Error bars represent exact (Clopper-Pearson) 95% confidence intervals

Moreover, no significant difference between CBCT and dMRI was noted for defect classification (sensitivity/ specificity: 86.5% (95% CI 0.780–0.926)/ 99.1% (95% CI 0.985–0.995) for CBCT, and 84.4% (95% CI 0.755–0.910)/ 99.1% (95% CI 0.985–0.995) for dMRI; *p* = *0.625*; Fig. 5). To account for the multifactorial structure of the data (modality, defect size, and wall type), classification accuracy was analyzed using Firth penalized-likelihood logistic regression. Again, no significant difference between modalities was observed (adjusted OR for dMRI vs. CBCT 0.85, 95% CI 0.38–1.88, *p* = 0.68), and this result was unchanged after additional adjustment for reader (OR 0.85, 95% CI 0.38–1.88, *p* = 0.68). Classification accuracy was significantly lower for small than for large defects (adjusted OR 0.43, 95% CI 0.18–0.97, p = 0.042), whereas wall count showed only a non-significant trend toward higher accuracy with an increasing number of walls (joint penalized likelihood-ratio p = 0.101).

**Fig. 6:**
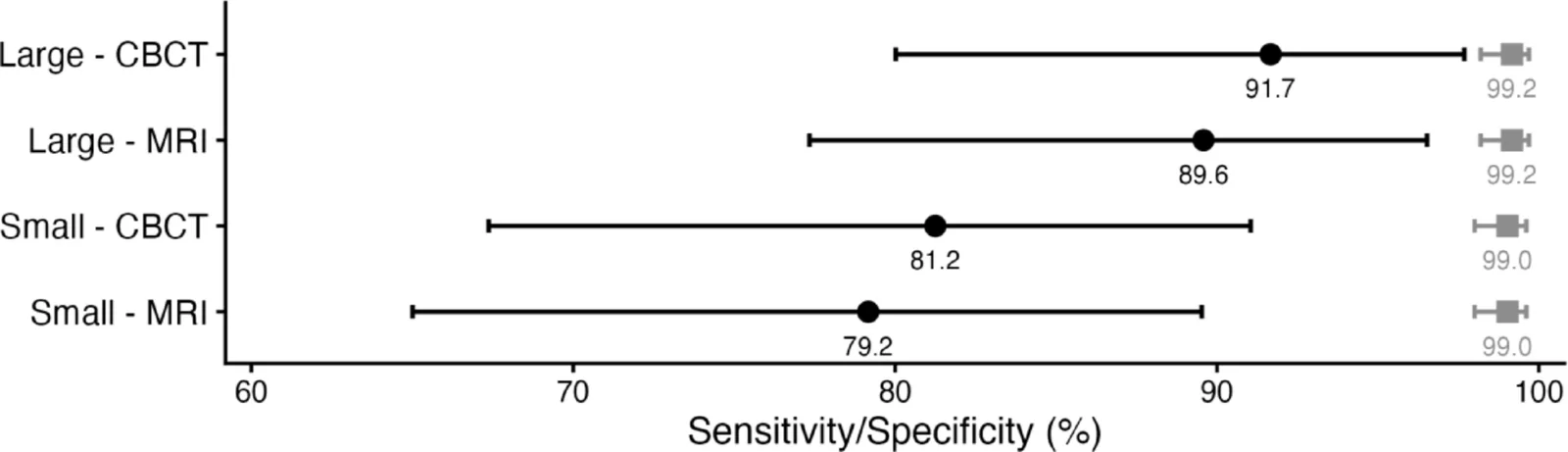
Sensitivity and Specificity (%) of CBCT and dMRI for detection and correct classification for small, respectively large peri-implant bone defects. Error bars represent exact (Clopper-Pearson) 95% confidence intervals

Consistent with this, classification sensitivity was greater for large than for small defects in both modalities, with specificity remaining comparable between the groups. For small defects, sensitivity and specificity were 81.3% (95% CI 0.674–0.911) and 99.0% (95% CI 0.980–0.997) for CBCT and 79.2% (95% CI 0.650–0.895) and 99.0% (95% CI 0.980–0.996) for dMRI. For large defects, sensitivity and specificity were 91.7% (95% CI 0.800–0.977) and 99.2% (95% CI 0.982–0.997) for CBCT and 89.6% (95% CI 0.773–0.965) and 99.2% (95% CI 0.982–0.997) for dMRI (Fig. 6). In CBCT, small 1-wall defects showed the lowest sensitivity at 75% (95% CI 0.428–0.945), whereas large 1-wall and large 4-wall defects achieved the highest sensitivity at 100% (both 95% CI 0.735–1.000). In dMRI, sensitivity was lowest for small 1-wall defects (58%, 95% CI 0.277–0.848), while small 4-wall and large 2-wall defects reached the highest sensitivity of 100% (95% CI 0.735–1.000; Table 1).

**Table 1:** Sensitivity (Se) and Specificity (Sp) in % of CBCT and dMRI for defect classification by defect type and size

	CBCT (%)				dMRI (%)			
	Small		Large		Small		Large	
	Se	Sp	Se	Sp	Se	Sp	Se	Sp
1-wall defect	75	100	100	99	58	100	83	99
2-wall defect	83	99	83	98	75	99	100	99
3-wall defect	83	99	83	99	83	98	92	98
4-wall defect	83	98	100	100	100	99	83	100

## Discussion

In this in vitro study, the reliability and accuracy of dMRI for detecting and classifying peri-implant defects around single titanium implants were found to be not significantly inferior to those of CBCT, even though as a trend CBCT performed superior to dMRI. While CBCT is a well-established gold standard for detecting peri-implant bone loss around titanium implants [36–38], we can show that novel dMRI coil and sequence optimization strategies offer a promising alternative to CBCT along with superior soft tissue contrast and no ionizing radiation. However, the current study focused exclusively on defect detection and classification under experimental conditions and did not investigate inflammatory activity, soft tissue pathology, or clinical outcomes. For the first time, it has been demonstrated that dMRI can visualize tissues at submillimeter distances adjacent to dental titanium implants. These findings are of clinical importance as peri-implantitis progresses in a non-linear and accelerating manner, making early detection essential to prevent irreversible damage and preserve implant stability [16]. Diagnostic imaging is essential for early peri-implant bone loss identification, particularly when clinical signs are still minimal. The classification of the defect morphology plays an important role in finding the adequate therapy [39].

The presence of titanium implants poses a significant challenge for MRI due to susceptibility artifacts caused by local magnetic field inhomogeneities [40]. One study demonstrated that embedding titanium implants in a 3% gelatin phantom and performing 3 Tesla MRI imaging resulted in pronounced signal voids caused by susceptibility artifacts, leading to an overestimation of implant size [41]. Another investigation found that pure titanium implants produced moderate artifacts in T1-weighted Fast-Spin-Echo sequences. In contrast, high-intensity artifacts appeared in T2-weighted Fast-Spin-Echo and Gradient-Echo sequences, which are particularly sensitive to metal-induced distortions. Factors influencing the severity of these artifacts include magnetic field strength, the choice of imaging sequences, and geometric parameters during imaging [28]. This study employed the compressed sensing SEMAC sequence, which represents an established technique in musculoskeletal imaging by combining slice encoding for metal artifact correction (SEMAC) and view angle tilting (VAT) to reduce both in-plane and through-plane distortions near metal implants [32, 33, 42].

Further strengths of this study include the use of an established bone model to mimic the human mandible with a complex simulation of the adjacent bone and soft tissue structures, as well as realistic peri-implant bone defects. Titanium was chosen as the implant material instead of zirconia, as used in previous studies, as it remains the most commonly used material for dental implants. Furthermore, an innovative and novel coil was employed to further improve the image quality. The use of an intraoral coil in this study significantly enhanced the signal-to-noise ratio, enabling higher-resolution imaging within clinically feasible scanning times [43]. Recent advancements in intraoral coils have achieved an isotropic voxel size as small as (0.21 mm)^3^ [44]. Additionally, the intraoral coil improved the visualization of anatomical details, particularly in regions close to the implant, where susceptibility artifacts are most pronounced [29, 45]. In general, the metal artifact suppression could also be combined with other intraoral coil concepts depending on the size and extend of the implant structure [46–48].

A previous approach to assess peri-implant bone formation using MRI was conducted by Korn et al. in a minipig model. The study demonstrated that MRI allows radiation-free visualization of peri-implant bone formation next to dental implants. However, the implants used were made of polyetheretherketone and coated only with a thin layer of titanium, which differs from the solid titanium implants typically used in clinical practice. Nevertheless, their findings suggest that MRI is a promising tool for monitoring osteointegration [49].

Next to IR, CBCT has traditionally been considered the gold standard for imaging peri-implant bone loss due to its high spatial resolution and ability to depict bone structures. However, its diagnostic performance is affected by factors such as voxel size, field of view, and beam-hardening artifacts [50]. Larger defects are reliably detected with CBCT, whereas smaller lesions often present challenges due to shadowing, scattered radiation, and streak artifacts [51]. A higher detection accuracy is observed for larger defects, underlining the impact of defect size on diagnostic performance [52]. The sensitivity (86.5%) and specificity (99%) observed for CBCT in this study are consistent with previously reported data on peri-implant bone defect detection and classification, further confirming its diagnostic reliability. Minor variations between the present findings and earlier studies may be attributed to differences in the experimental setup, such as defect size distribution, imaging protocols, or assessment criteria.

Schwindling et al. [36] analyzed 72 datasets with 1-wall, 2-wall, 3-wall, and 4-wall defects around titanium implants placed into bovine ribs. High definition CBCT achieved a sensitivity of 96% and a specificity of 99%, while low-dose CBCT showed a sensitivity of 93% and a specificity of 98%. The lowest diagnostic accuracy was reached for small 1-wall defects, with sensitivity and specificity values of 92% and 99% for both CBCT modalities. In the present study, dMRI achieved a sensitivity of 84% and a specificity of 99%, allowing for a direct comparison with CBCT settings. Kuhl et al. [5] investigated 2-wall, 3-wall, and 4-wall defects measuring 1 and 3 mm around titanium implants placed into an edentulous human lower jaw with a sample size of six specimens. Sensitivity values were 68% for small defects and 79% for larger defects, with a specificity of 25% in both groups. Another study evaluated 80 implants that were placed into bovine ribs, where the bone surrounding the implant sites was eroded using perchloric acid for up to 12 h. Depending on the field of view, sensitivity in CBCT ranged from 68 to 80% and specificity from 67 to 81% [7, 53].

This study has several limitations. First, the investigation was performed under controlled in vitro conditions using bovine rib specimens with artificially created peri-implant defects. Although this approach allowed for a fair comparison of imaging modalities, and although the use of perchloric acid etching enabled the creation of irregular peri-implant bone surfaces, it does not fully replicate the biological and anatomical complexity of peri-implant disease in clinical practice. Factors such as patient motion, anatomical variability, and inflammatory soft and hard tissue alterations were either absent or only partially represented. Furthermore, the predefined defect prevalence and standardized defect morphology may have introduced spectrum bias and do not reflect the diagnostic uncertainty encountered in routine clinical setting. Consequently, the present findings should be interpreted as evidence of diagnostic performance under standardized experimental conditions rather than as direct evidence of clinical performance. The relatively small sample size (n = 48 implants) limits the statistical power of the analyses. As a result, the study should be considered as an exploratory and hypothesis-generating pilot study.

Moreover, the implants were covered with muscle tissue, a factor often overlooked in other studies but essential for simulating realistic clinical conditions [52]. This approach ensures, that the findings are not only reproducible but also clinically applicable. Moreover, the use of a MRI sequence with non-isotropic voxels in this study limits the ability to perform volumetric reconstructions, unlike CBCT datasets, which may explain the non-detection of certain, particularly small, defect types. In future studies, the use of MRI sequences with isotropic voxels and artifact reduction, such as MAVRIC-SL or MSVAT-SPACE, could be investigated [54, 55]. Similarly, SEMAC 2D sequences could be adjusted to yield isotropic voxels.

Compared to CBCT, MRI-based dental imaging is associated with longer acquisition times, higher system and operational costs, and currently limited workflow integration in routine dental practice. In addition, the availability of intraoral coils as well as specialized, metal artifact-reducing sequences remains restricted. The limited field of view of intraoral coils further necessitates multiple acquisitions for full-jaw assessment, which may reduce efficiency in clinical workflows. Altogether, these factors currently limit the broad clinical applicability of dMRI.

Additional in vivo studies are necessary to validate these results in a clinical setting, with particular attention to patient motion. While MRI provides radiation-free imaging and superior soft tissue contrast, its higher cost and limited availability compared to CBCT remain challenges. However, the introduction of novel dedicated MRI systems specifically designed for dental applications [56] is likely to address these limitations soon. Despite these limitations, our findings demonstrate the promising potential of SEMAC and CS-SEMAC sequences for improving dental MRI. Future research can build on these results to further refine imaging protocols and enhance clinical applicability.

## Conclusion

Under ex vivo conditions, optimized dMRI demonstrated a similar reliability as CBCT for peri-implant bone defects identification and classification around single titanium implants. Diagnostic accuracy showed a trend toward higher values for CBCT compared with dMRI; however, this difference did not reach statistical difference. Novel MRI sequence and coil strategies enabled improved visualization of peri-implant structures under controlled experimental conditions, suggesting potential for radiation-free three-dimensional assessment of peri-implant tissues in the future. However, these findings are limited to an in vitro setting, and further in vivo studies are required to evaluate clinical applicability and diagnostic performance under real-world conditions.

## Data Availability

The datasets used and analyzed during the current study are available from the corresponding author on reasonable request.

## Abbreviations

CBCT: Cone-beam computed tomography
cs: Compressed sensing
dMRI: Dental magnetic resonance imaging
IR: Intraoral radiography
*κ*: Cohen’s Kappa
OR: Odds ratio
SEMAC-VAT: Slice encoding for metal artifact correction - View angle tilting
1w: 1-Wall
2w: 2-Wall
3w: 3-Wall
4w: 4-Wall
